# Unmet needs and quality of life of adult patients while receiving cancer treatment in Uganda: A cross-sectional study

**DOI:** 10.1371/journal.pone.0339827

**Published:** 2025-12-31

**Authors:** Joshua Kanaabi Muliira, Allen Naamala Mayanja, Prossy Nandawula, Jane Frances Anyango, Scovia Nalugo Mbalinda

**Affiliations:** 1 Department of Adult Health and Critical Care, College of Nursing, Sultan Qaboos University, Muscat, Oman; 2 Allen Naamala Mayanja, Uganda Cancer Institute, Ministry of Health, Kampala, Uganda; 3 Prossy Nandawula, Lecturer, School of Health Sciences, Soroti University, Soroti, Uganda; 4 Jane Frances Anyango, Assistant Professor of Nursing, School of Nursing, Ball State University, Muncie, Indiana, United States of America; 5 Department of Nursing, School of Health Sciences, College of Health Sciences, Makerere University, Kampala, Uganda; Universitas Pendidikan Ganesha, INDONESIA

## Abstract

**Introduction:**

The increasing burden of cancer in resource-limited settings like Uganda puts pressure on healthcare systems and leaves many patients’ needs unmet. These unmet needs impact service uptake, perceptions of care, and health outcomes. However, the extent and effect of unmet care needs on patients’ health outcomes remain poorly characterised.

**Methods:**

A cross-sectional study involved 170 adult cancer patients at the Uganda Cancer Institute (UCI). Participants filled out validated instruments such as the Hospital Anxiety and Depression scale (HADS), the EORTC QLQ-30 (Quality of life scale) and the Supportive Care Needs Survey (SCNS-SF34). Descriptive statistics, correlation and regression analyses examined the relationship between unmet needs and health outcomes.

**Results:**

Nearly half (45.9%) of participants reported clinically significant anxiety symptoms, while 35.9% had depressive symptoms. The highest unmet needs were in physical/daily living (mean = 73.59 ± 20.26), psychological (51.77 ± 18.24), and health system/information domains (38.96 ± 19.59). Poor quality of life was observed in role (18.14 ± 27.01) and social functioning (9.41 ± 18.48). Fatigue (β = 0.23, p < 0.01) and anxiety (β = 0.20, p < 0.01) were the strongest predictors of overall unmet needs.

**Conclusion:**

Cancer patients in Uganda experience substantial unmet needs, particularly in physical, psychological, and informational support, which are strongly linked to worse mental health and quality of life. To improve patient outcomes, integrated interventions targeting symptom management, psychosocial support, and health system strengthening are urgently needed.

## Introduction

It is estimated that by the year 2040, there will be 28.4 million new cancer cases globally, with the majority (65% to 95%) being in developing countries due to the high burden of risk factors and demographic, lifestyle, and epidemiological transitions [1]. The burden of cancer morbidity and mortality is evident in resource-limited settings such as those in Eastern Sub-Saharan Africa, since some have the world’s highest fatality rates (75.5%) for breast cancer [2]. Uganda, a country of 47 million people, is in Eastern Sub-Saharan Africa and has a cancer case fatality rate of 80% [2]. Ugandans are estimated to have a cancer age-standardized incidence rate and death rate of 200 per 100000 and 150 per 100000, respectively [2]. In 2018, approximately 60000 Ugandans had cancer and needed specialized care, but only 5% had access to medical services at the country’s only comprehensive cancer treatment centre, the Uganda Cancer Institute [3].

The number of Ugandans with undiagnosed cancer due to a lack of access to health care is estimated to be much higher. Reports from the Uganda Cancer Registry show that cancer incidence is rapidly increasing, especially cancer of the prostate, breast, cervix, oesophagus, colorectum, and liver [4]. Other reports have highlighted an association between quickly rising incidences of cancer and HIV/AIDS [3]. This growing burden of cancer in Uganda is taking place on the backdrop of a cancer care system with challenges, such as the limited capacity for cancer prevention and early detection [5], late presentation and diagnosis, and lack of access to cancer treatments [6] and other resources. For instance, the country is experiencing persistent shortages of blood products for cancer patients [7–9], and lacks formal programs to train nurses in oncology, supportive care, and psychosocial care for cancer patients [10–12].

Emerging evidence from Uganda and other sub-Saharan African countries has begun to characterise the multifaceted unmet supportive care needs among cancer patients. Nakaganda and colleagues (2021) highlighted the critical health system and social challenges impeding adequate cancer care, emphasising gaps in patient education and psychosocial support [13]. Katende and Nakimera (2017) documented high levels of anxiety and depression among caregivers in Ugandan cancer care settings, pointing to the psychosocial distress linked to unmet needs [14]. Similarly, Muliira and Kizza (2019) underscored the profound emotional and physical burdens borne by family caregivers of adult cancer patients, adding complexity to patient care dynamics in sub-Saharan Africa [15]. Moreover, Bray and colleagues (2022) presented a comprehensive overview of the cancer burden in sub-Saharan Africa, and called attention to the major data gaps and unmet health system needs that persist throughout the cancer care continuum [16].

Collectively, these studies reveal that unmet needs among cancer patients in these contexts encompass aspects related to physical symptom management, psychological and emotional support, informational needs, and social care, all of which often exacerbated by resource constraints and stigma [13–15] Despite these insights, there remains a paucity of comprehensive, quantitative assessments of unmet supportive care needs across diverse cancer types in Uganda and Africa at large, particularly using validated instruments. Thus, characterisation of these needs is essential to inform the development of culturally appropriate interventions to improve the quality of life and treatment outcomes of cancer patients.

The inadequacies of the cancer care system generate dissatisfaction among patients with cancer and their caregivers. For instance, many patients with breast cancer are unsatisfied with the cancer care services and instead resort to complementing mainstream therapy with herbal medicines and native healers [17]. Concurrent use of non-prescribed herbal medications with cancer treatments such as chemotherapy can lead to fatal drug interactions, treatment failure, and adverse effects [18]. Additionally, avoiding chemotherapy during the last 30 days of life has become a goal of cancer care in countries with established cancer care systems in North America, Asia, and Europe [19]. In Uganda, due to late presentation and diagnosis, many patients (45%) receive chemotherapy in the last thirty days of life, and non-clinical factors still drive chemotherapy treatment decisions [19]. These tendencies are associated with low chemotherapy efficacy, and increased patient and caregiver supportive care needs due to end-of-life chemotherapy [19].

The inadequacies may also be contributing to the high symptom burden and suboptimal symptom management among cancer patients. On average, Ugandan cancer patients report 18 symptoms, the most prevalent being pain, sexual problems, weight loss, chronic fatigue and poor self-image [20]. Moreover, stigma is another major hindrance to cancer care and a significant unmet need for patients and their caregivers. A qualitative study showed that Ugandan patients with breast cancer perceived and internalized stigma associated with breast cancer, leading to delayed care-seeking activities and low treatment completion rates [21]. The participants pointed out that stigma may be overcome by providing social support and patient education to increase understanding of the disease [21].

The above background shows that in Uganda, unmet needs could be severely impacting the health outcomes of patients with cancer. However, high-quality patient-and-family-centred care can only be achieved after addressing the unmet needs. Cancer care that minimally addresses the unmet needs of patients results in mismatched healthcare, which tends to increase non-adherence to treatment, healthcare expenditures, and harmful effects (Wen & Gustafson, 2004). This aligns with the Supportive Care Needs framework, which posits that unmet needs arise from gaps in physical, psychological and systematic care [22]. While Ddungu and colleagues(2018) focused on Metastatic breast cancer [7], this study examines unmet needs across all types of cancer, providing a broader perspective*.* This baseline study of the unmet needs of cancer patients may help guide interventions to enhance the capacity for cancer supportive and psychosocial care. Understanding Uganda’s most impactful unmet needs can facilitate the development of affordable and culturally relevant interventions that adequately support the population.

The study aimed to identify unmet needs affecting cancer patients’ health outcomes in Uganda to guide the development of a nurse-led supportive care intervention program, focusing on depressive symptoms, anxiety, and quality of life.

## Materials and methods

### Study design

The study used a descriptive cross-sectional design to collect data from a sample of patients admitted at the Uganda Cancer Institute (UCI) for cancer treatment. The design enabled prompt quantitative data collection to generate a foundation to support designing of intervention studies.

### Study participants and setting

The study focused on Ugandan adult patients receiving treatment for cancer of any type at the UCI. The participants were individuals over 18 years diagnosed with any cancer. The patients were recruited for the study during admission to the UCI (Kampala branch). UCI is a public cancer care organization under Uganda’s Ministry of Health and focuses on research, training, consultation, prevention, and cancer treatment for paediatric and adult patients. The Kampala branch is the largest cancer treatment centre in the country. It maintains an inpatient facility with a capacity of 80 beds and attends to an average of two hundred patients per day. UCI is a national referral and teaching hospital.

### Study sample

A priori was conducted using G*Power version 3.1.9.7 to determine the minimum sample size required for multiple linear regression analysis [23]. The initial power calculation considered a model with up to ten independent variables. These candidate predictors were selected based on prior literature and theoretical relevance to unmet needs, including demographic variables (age, gender, education, social status or employment status), clinical factors (ECOG performance status, stage of cancer), and key symptom variables (fatigue, pain, anxiety, and depression). This number of predictors was chosen to balance model complexity with statistical power and to avoid overfitting, given the sample size. During the analysis phase, only those variables demonstrating significant bivariate correlations with unmet needs were retained for inclusion in the final regression models. This resulted in testing eight predictors in the final models. The selection approach was pre-specified to ensure transparency and align with standard analytical practices for regression modelling. The rationale for each candidate predictor’s inclusion was summarized based on their relevance in the context of unmet needs among cancer patients.

The required minimum sample to achieve a minimum power of 80% for detecting a medium effect, a significance criterion of alpha (α) of.05, was estimated to be 136. After factoring in a 20% non-response rate, the required sample was estimated at 163. The study was able to recruit a sample of 170 patients with cancer (N = 170). A consecutive sampling technique was used to generate a sample of patients available during the study period. This technique was appropriate due to the lack of a comprehensive list of the accessible participants necessary to establish a sampling frame or a database of patients or a registry, as well as the need for real-time recruitment in a clinical setting*.*

The patients who met the inclusion criteria were recruited consecutively until the required sample size was obtained. The inclusion criteria were age of ≥ 18 years; a confirmed diagnosis of any cancer; actively receiving cancer care or therapy; a cancer diagnosis for at least the past 3 months; and a functional status adequate to lead to informed consent and clear responses to the questionnaire. The patients were excluded if they were not citizens of Uganda (n = 15) and had concurrent morbidity with a neurological disorder (n = 8). The non-citizens were excluded to minimize cultural/linguistic heterogeneity in unmet need reporting.

### Study instrument

The measured variables included the patient demographic and clinical characteristics, health outcomes (anxiety, depressive symptoms, and quality of life), and unmet needs. Data were collected via interviewer-administered questionnaires. The questionnaire was written in English (the official language used in Uganda and during healthcare delivery in all healthcare facilities) and the local language (Luganda) for participants who do not speak English. The questionnaire was translated into Luganda following a rigorous process to ensure linguistic and cultural appropriateness. Two bilingual language experts from the Institute of Languages, experienced in health terminology, performed the translation. We adhered to World Health Organization (WHO) guidelines for translation and adaptation, which included forward translation, back-translation, and iterative reconciliation to achieve semantic and conceptual equivalence.

To assess cultural equivalence and clarity, cognitive interviews were conducted with 20 pilot participants. During these interviews, participants were asked to explain the meaning of selected questionnaire items in their own words and to identify any confusing terms. Feedback obtained led to minor wording modifications to enhance comprehension while preserving the original meaning. The pilot testing also evaluated cultural appropriateness and relevance of the questionnaire items to the experiences of Ugandan cancer patients, ensuring that the instrument accurately reflected the local context. The Luganda questionnaire was used to collect data from 60 (35.3%) participants.

The questionnaire consisted of four sections, i.e., demographic and clinical characteristics, the Hospital Anxiety and Depression Scale (HADS), the Quality of Life Cancer Patients Scale (EORTC-QLQ-C30), and the Supportive Care Need Survey short form (SCNS-SF 34). The demographic characteristics of cancer patients include aspects such as gender, age, marital status, level of education and others, the clinical characteristics included factors such as the date of cancer diagnosis, type of cancer, type of current cancer treatment, current symptoms or complaints, pain rating, and ECOG performance status (the Eastern Cooperative Oncology Group (ECOG) scale ranks the patient’s ability to function and ability for selfcare). The patients’ health outcomes considered were psychological well-being and QoL, measured using the HADS and the EORTC-QLQ-C30. The unmet needs were measured with the SCNS-SF-34.

The HADS is an emotional distress self-report questionnaire, and it is one of the most frequently used in oncology. The HADS has two subscales to detect anxious (HADS-A) and depressive (HADS-D) symptoms. Each subscale consists of seven items with a 4-point ordinal response format, and the total scores range from 0 to 21 in each subscale, with higher scores indicating higher levels of anxious or depressive symptoms [24]. Participants answered each item thinking of how they felt and behaved during week [24]. The internal consistency reliability, assessed with ordinal alpha, for the HADS-A (0.92) and HADS-D (0.88) shows good reliability [25]. The Cronbach’s alpha for HADS-A and HADS-D ranges from 0.87 to 0.93 [25]. The Cronbach’s alpha for HADS-A and HADS-D in the current study was 0.71 and 0.56, respectively. The lower Cronbach’s alpha for HADS-D (α = 0.56) may reflect cultural stigma around reporting depressive symptoms. Thus, future studies should consider validating the HADS in large and more diverse samples of Ugandan cancer patients.

The EORTC QLQ-C30 consists of 30 items divided into five domains (physical, role, emotional, cognitive, and social), six symptom scales (dyspnea, insomnia, loss of appetite, constipation, diarrhoea, and the perceived financial impact of the disease), and a global health and quality-of-life scale [26]. All items are scored on a four-point Likert scale ranging from 1 to 4 (not at all, a little, quite a bit, and very much), except the two items on the global health and quality of life scale. The two items are scored on a seven-point Likert scale ranging from 1 (very poor) to 7 (excellent). The raw scores of the EORTC QLQ-C30 are transformed into 0–100-point scales, with higher scores representing a higher quality of life for functional scales (physical, role, emotional, cognitive, and social function). The higher scores on the symptom scale represent higher symptom burden. The EORTC QLQ-C30 has been used in Uganda in previous studies and found to have acceptable Cronbach’s alpha values ranging from 0.79 to 0.96, [27]. The Cronbach’s alpha of the EORTC QLQ-C30 in the current study was 0.84.

The SCNS-SF-34 assesses the unmet needs in cancer patients across five domains of psychological, health systems and information, physical and daily living, patient care and support, and sexuality [28]. The SCNS-SF-34 has been used in various countries and cultures and is stable and reliable [29]. The Cronbach’s alpha coefficients for the five factors range from 0.86 to 0.96 [28,30]. The patients rate their need for help in the past month using a 5-point Likert scale (1= = no need, not applicable; 2 = no need, satisfied; 3 = low need; 4 = moderate need; 5 = high need). The items are summed to generate a total for each domain. The domain scores are converted to standardised Likert summated scores, ranging from 0 to 100, with higher scores indicating a higher perceived unmet care need^24^. The Cronbach’s alpha of the overall SCNS-SF-34 in the current study was 0.91, and the Cronbach’s alphas of its domains are presented in Table 3. The original authors granted permission to use the HADS, SCNS-SF34, and the EORTC-QLQ-C30.

**Table 3:** Unmet needs of adult patients with cancer in Uganda (N = 170).

Domain of Unmet Needs	Number Items	Cronbach’s Alpha	Standardized Summated Likert Scores		
			Min	Max	Mean ± SD
Overall Unmet Needs	34	0.91	18.38	89.71	46.29 ± 12.83
Physical and daily living needs	5	0.85	5	100	73.59 ± 20.26
Psychological needs	10	0.84	13	100	51.77 ± 18.24
Health system and information needs	11	0.93	18	100	38.96 ± 19.59
Patient care and Support needs	5	0.80	15	95	35.24 ± 16.12
Sexuality needs	3	0.78	0	99.96	27.73 ± 19.89

Min, minimum score; Max, maximum score; SD, standard deviation.

### Ethical considerations

The study was reviewed and approved by the Institutional Review Board of Ball State University (IRB protocol # 2122231−1), UCI (UCI-2023–83) and the Uganda National Council for Science and Technology (HS4842ES). After ethical approval, permission was sought from the administration of UCI to advertise the study in all units. Data collection was initiated after the research assistants (two registered nurses) underwent training focusing on confidentiality, consent process, privacy, bias, and protection of participants’ information. The eligible participants were required to complete the consent process before data collection. The participants were informed of their freedom to stop participating at any time without any penalty.

Codes were used to identify the filled questionnaires. Data was collected in a private room in the selected units within the UCI facilities. The participants were assured that all information collected was only for study purposes, and results would be presented as group data with no possibility of identifying individuals’ responses. The study activities did not involve any invasive procedures. Discussing topics related to cancer could trigger incidents or emotional reactions requiring attention by a trained healthcare professional, but there were no such occurrences in this study.

### Data collection procedure

The research assistants (RAs) conducting data collection interviews were registered nurses with competencies in patient education and explanation of health-related concepts. After obtaining ethical approval, the investigators recruited two registered nurses to work for the project. The RAs were oriented and trained in a workshop focusing on the study purpose, participant eligibility criteria, selection, confidentiality, privacy, autonomy, bias, and consent process during research. The investigators-initiated contact with the heads of the units/departments at UCI, provided copies of IRB approval letters, and verbally explained the study purpose and plan. The meetings resulted in permission and support to recruit participants.

The RAs (under the investigators’ supervision) then pre-tested the study questionnaire on twenty patients with cancer. The pre-test provided insight into the time needed to administer the questionnaire, the clarity of the items, and the best logical sequencing of the scales. No significant adjustments were made to the questionnaire. Pre-test data were not included in the final analysis.

The RAs initiated data collection with the finalized questionnaire from 22^nd^ September 2024–20^th^ December 2024. On data-collection days, RAs were assigned to different units daily. The RAs introduced themselves to the unit/department in charge. Once on the unit, the RAs requested nurses to identify patients who met the inclusion criteria. The nurses’ assistance was crucial because they spent a substantial amount of time with the patients at the facility and got to know them. The RAs approached eligible participants to explain the study’s purpose and the extent of participation. Those willing to participate in the study were provided privacy to consent and start the data collection process. The RAs read the items on the questionnaire to the participants and allowed time for them to select the most appropriate response. This approach helped to reduce participant exhaustion and fatigue. Some participants could read and complete the questionnaire themselves (74%). Collecting data from each patient required an average of 56 ± 7.83 minutes. Each questionnaire was checked immediately for completeness, and clarifications were sought before leaving the data collection point.

### Data analysis

The data was analysed using SPSS version 29. Cronbach’s alphas were calculated to assess the internal consistency reliability of the scales used to measure unmet needs, QoL, and anxiety and depressive symptoms. Descriptive statistics were used to summarize the unmet needs, QoL, anxiety, and depression symptoms. Following standard analytical procedures, we first examined bivariate correlations to identify variables significantly associated with unmet needs. Only variables demonstrating significant bivariate associations (p ≤ 0.05) were included in the multivariable regression models. This approach was used to ensure model parsimony and to reduce multicollinearity and overfitting risks given the sample size. Due to sample size limitations and data availability, it was not feasible to adjust for all potential confounders. This variable selection process is fully described here to provide transparency in the modeling strategy.

Additionally, the stepwise method of multiple linear regression analysis was used. Stepwise regression is used to select the best combination of independent variables for a regression model by adding or removing them based on statistical criteria, creating a simpler, more parsimonious model that risks less overfitting and is easier to interpret, especially when dealing with a large pool of potential predictors. The significance level for all the statistical tests was set at *p ≤ *0.05, and all point estimates are expressed with confidence intervals.

## Results

### Descriptions of the sample of cancer patients

The sample consisted of 170 patients. A total of 234 were approached to participate, and 170 (72.7%) agreed and consented to participate. Table 1 presents a summary of the demographic characteristics of the participants. Most patients had only primary level education (66.5%) and were diagnosed with cancer at a young age (mean = 48.87 ± 14.12 years). On average, the patients participated in the study after 29.73 ± 16.68 months, from when the cancer was first diagnosed, and therefore, had a good experience with their unmet needs. The common types of cancer were cervical (37.7%), breast (21.8%), oesophageal (7.1%), ovarian (4.1%), and prostate cancer (4.1%).

**Table 1:** Demographic characteristics of the patients with cancer (N = 170).

Characteristic	Category	n(%)
Gender	Male	28 (16.5)
	Female	142 (83.5)
Marital status	Married	85(50)
	Single	56 (32.9)
	Divorce/separated/Widowed	29 (17.1)
Highest level of education	Primary school	113 (66.5)
	Secondary school	43 (25.3)
	Post-secondary	6 (3.5)
	≥Bachelor’s degree	8 (4.7)
Employment status	Retired	40 (23.5)
	Not employed	46 (27.1)
	Not working due to illness	20 (11.8)
	Employed/actively working	64 (37.6)
Age in years (Mean = 49.87 ± 14.12)	18–40	46 (27.1)
	41–60	84 (49.4)
	≥61	40 (23.5)
Time (months) since cancer was first diagnosed (Mean = 29.73 ± 16.68)	3–6	14 (8.2)
	7–12	17 (10)
	≥13	139 (81.8)
Site of the cancer disease	Cervix	64 (37.7)
	Breast	37 (21.8)
	Esophagus	12 (7.1)
	Prostate	7 (4.1)
	Ovary	7 (4.1)
	Stomach	5 (2.9)
	Bone	5 (2.9)
	Kaposi’s sarcoma	5 (2.9)
	Ear/Nose/Throat	4 (2.4)
	Skin	4 (2.4)
	Leukemia	4 (2.4)
	Tongue and Gum	3 (1.8)
	Eye	3 (1.8)
	Colon	2 (1.2)
	Vulva/Brain/Bladder/Anus/	8(4.7)
	Liver/Lung/Lymphoma/Uterus (one patient each)	

The clinical characteristics of the participants (self-report) are presented in Table 2. Most of the patients reported that they were initially diagnosed with stage 3 (29.4%) or stage 4 (37.6%) cancer, chronic pain (72.4%), and were ambulatory (31.8%) or ambulatory with restrictions (31.8%) according to the ECOG score. Many patients were receiving chemotherapy (42.4%) or a combination of chemotherapy and radiation therapy (24.7%). Most participants had moderate to severe pain daily (55.9%) and at the time of data collection (56.5%). The most distressing symptoms were pain (86.5%), general weakness (22.9%), vomiting (20.6%), lack of appetite (19.4%), bleeding (14.1%), and nausea (12.4%).

**Table 2:** Clinical characteristics of patients (N = 170).

Characteristic	Category	n (%)
ECOG performance score (Mean = 1.44 ± 0.71)	0	7 (4.1)
	1	96 (56.5)
	2	54 (31.8)
	3	12 (7.1)
	4	1 (0.6)
Stage of cancer disease	Stage 1	18 (10.6)
	Stage 2	50 (29.4)
	Stage 3	64 (37.6)
	Stage 4	31 (18.2)
	Cannot be staged	7 (4.1)
Current type of cancer treatment	Palliative care	22 (12.9)
	Radiotherapy	23 (13.5)
	Chemotherapy	72 (42.4)
	Chemotherapy and Surgery	5 (2.9)
	Radiotherapy and Chemotherapy	42 (24.7)
	Radiotherapy, Chemotherapy, & Surgery	6 (3.5)
Do you have chronic pain?	Yes	123 (72.4)
	No	47 (27.6)
Pain rating now on a scale of 0–10 (Mean = 4.11 ± 2.48)	Mild (0–3)	74 (43.5)
	Moderate (4–6)	67 (39.4)
	Severe (7–10)	29 (17.1)
Daily pain on scale of 0 −10 (Mean = 4.32 ± 2.48)	Mild (0–3)	75 (44.1)
	Moderate (4–6)	66 (38.8)
	Severe (7–10)	29 (17.1)
Most disturbing symptoms today (Multiple Responses)	Pain	147 (86.5)
	Nausea	21 (12.4)
	Vomiting	35 (20.6)
	Bleeding	24 (14.1)
	Lack of appetite	33 (19.4)
	Fever	17 (10)
	General weakness	39 (22.9)
	Loss of hair	19 (11.2)
	Inability to move.	4 (2.4)
	Tiredness	18 (10.6)
	Wounds	22 (12.9)
	Lack of sleep	3 (1.8)
	Tired of the hospital	2 (1.2)

### The unmet needs of adult cancer patients in Uganda

The results presented in Table 3 show that overall unmet needs of the patients were moderate (Mean = 46.29 ± 12.83) on a scale of 0–100. However, the patients had very high levels of unmet needs (using standardized mean scores) related to physical and daily living, psychological needs, health systems and information needs. The lowest level of unmet needs were those related to sexuality. The SCNS-SF34 and its respective domains were reliable in measuring the unmet needs, considering the Cronbach’s alpha ranging from 0.78 to 0.93.

### Psychological well-being and quality of life of the patients

Table 4 shows the results of the patients’ psychological well-being and QoL measured using the HADS and the EORTC-QLQ-C30. Many patients had symptoms equivalent to abnormal levels of anxiety (45.9%) and depressive (35.9%) symptoms. The mean score for anxiety and depression symptoms shows that the sample had borderline symptoms. All the QoL scales and single-item measures were transformed into standardized scores ranging from 0 to 100. A high score for a functional scale represents a high healthy level of functioning, and a high score for the global health status represents a high QoL. However, a high score for a symptom scale (items) represents a high symptomatology or problems. The patients reported good global health status out of 100 points (Mean = 91.28 ± 17.50). However, the patients had low QoL in aspects such as cognitive function (43.63 ± 28.27), role function (18.14 ± 27.01), and social function (9.41 ± 18.48). The most problems or problematic symptoms were financial difficulties, pain, fatigue, insomnia, appetite loss, constipation, and dyspnoea (Mean score ≥ 40.00). The patients reported no other symptoms except those documented in Table 4.

**Table 4:** Description of the psychological well-being and quality of life of patients.

Psychological Wellbeing	Category	n(%)	Mean± SD
Anxiety symptoms Anxiety symptoms	Normal (0–7)	50 (29.4)	9.85 ± 4.46
	Borderline (8–10)	42 (24.7)	
	Abnormal (11–21)	78 (45.9)	
Depression symptoms	Normal (0–7)	50 (29.4)	9.31 ± 3.43
	Borderline (8–10)	59 (34.7)	
	Abnormal (11–21)	61 (35.9)	
Quality of Life			Mean± SD (Standardized Summated Scores)
QoL Global	Global Health status		91.28 ± 17.50
QoL Functional scales	Emotional function		57.45 ± 25.80
	Physical function		50.78 ± 20.76
	Cognitive function		43.63 ± 28.27
	Role function		18.14 ± 27.01
	Social function		9.41 ± 18.48
QoL Symptom scales	Financial difficulties		92.35 ± 18.50
	Pain		72.55 ± 25.15
	Fatigue		61.63 ± 24.60
	Insomnia		54.90 ± 34.48
	Appetite loss		53.14 ± 38.97
	Constipation		41.37 ± 43.75
	Dyspnea		40.20 ± 39.37
	Nausea and vomiting		2.96 ± 38.59
	Diarrhea		2.91 ± 37.73

QoL, Quality of Life; SD, standard deviation.

### Relationship between patient-reported unmet needs and health outcomes

Table 5 shows each category of psychological wellbeing (anxiety and depression symptoms) and aspect of QoL that were significantly associated with each type of unmet need at the bivariate level, using Pearson’s correlations. The results presented in Table 6 (linear regression analysis stepwise method) identify the factors with the most influence on each type of unmet need. The overall level of unmet needs was most significantly associated with fatigue (p < .01) and anxiety (p < .01), and the two factors contributed 22.9% to the variance in overall unmet needs.

**Table 5:** Correlation between unmet needs, psychological wellbeing, and quality of life (N = 170).

Type of unmet need	Factor	r	p-value	Type of unmet need	Factor	r	p-value
Overall unmet needs	Anxiety score	0.282	.000	Psychological needs	Anxiety	0.474	.000
	Depression score	0.163	.034		Depression	0.328	.000
	Pain	0.255	.001		Pain	0.19	.013
	Global health	0.201	.009		Fatigue	0.303	.000
	Fatigue	0.413	.000		Insomnia	0.172	.025
	Appetite loss	0.198	.001		Diarrhoea	0.163	.035
	Diarrhoea	0.234	.002		Nausea and vomiting	0.188	.014
	Financials difficulties	−0.154	.044		Role function	−0.171	.026
	Nausea and vomiting	0.237	.002		Physical function	−0.336	.000
	Insomnia	0.154	.045		Emotion function	−0.286	.000
	Physical function	0.324	.000		Cognitive function	−0.224	.003
	Emotional function	0.218	.004		Social function	−0.186	.015
	Social function	0.22	.004				
Health system and information needs	Fatigue	0.296	.000	Physical and daily living needs	Anxiety	0.397	.000
	Physical function	−0.185	.016		Depression	0.171	.025
Patient care and support needs	Pain	0.211	.006		Pain	0.316	.000
	Fatigue	0.154	.045		Global health	0.293	.000
	Physical function	−0.221	.004		Fatigue	0.369	.000
Sexual needs	Fatigue	0.173	.024		Appetite loss	0.295	.000
	Anxiety s	0.195	.011		Diarrhoea	0.283	.000
	Emotional function	−0.203	.008		Financials difficulties	0.166	.031
					Nausea and vomiting	0.241	.002
					Dyspnoea	0.251	.001
					Insomnia	0.363	.000
					Role function	−0.287	.000
					Physical function	−0.232	.002
					Emotional function	−0.172	.025
					Cognitive function	−0.324	.000
					Social function	−0.346	.000

**Table 6:** Predictors of specific types of cancer patients’ unmet needs (N = 170).

Factors		Unstandardized		Standardized Coefficient	t	p- value	95%CI
		B	SE	B			
Overall unmet needs	Constant	26.99	2.91	0.39	9.27	.000	21.24 - 32.74
	Fatigue	0.20	0.04	0.23	5.60	.000	0.13 - 0.28
	Anxiety	0.68	0.20		3.39	.001	0.28–1.07
Health system & Information needs	Constant	24.42	3.89	0.30	6.27	.000	16.73–32.10
	Fatigue	0.24	0.06		4.02	.000	0.12–0.35
Sexual needs	Constant	36.71	3.67	−0.20	10.01	.000	29.47–43.94
	Emotional functioning	−0.16	0.06		− 2.68	.008	−0.27 - - 0.04
Patient care & support needs	Constant	43.95	3.20	−0.22	13.72	.000	37.63–50.28
	Physical functioning	−0.17	0.06		− 2.94	.004	−0.29 - - 0.06
Psychological needs	Constant	33.71	6.45	0.40	5.22	.000	20.97–46.45
	Anxiety	1.63	0.28	0.20	5.83	.000	1.08–2.19
	Fatigue	0.15	0.05	−0.16	2.80	.006	0.04–0.25
	Physical functioning	−0.14	0.06		−2.15	.033	−0.27 - −0.01
Physical & daily living needs	Constant	52.36	5.14	0.28	10.19	.000	42.22–62.51
	Anxiety	1.30	0.31	0.21	4.21	.000	0.69–1.91
	Fatigue	0.18	0.06	−0.18	2.93	.004	0.06 −0.30
	Social functioning	−0.20	0.08	−017	−2.72	.007	−0.35 - - 0.06
	Role functioning	−0.13	0.05	0.15	−2.55	.012	−0.23 - - 0.03
	Diarrhoea	0.08	0.04		2.14	.034	0.01 −0.16

Fatigue was the only significant predictor of health system and information unmet needs (p < 0.01), responsible for 8.8% of the variance in this type of need. The sexual needs were most significantly associated with the level of emotional function (p < 0.01), and emotional function was responsible for 4.1% of the variance in sexual needs. The most significant predictors of patient care and support needs were the patient’s level of physical functioning (p < 0.01), and this factor was responsible for 4.9% of the variance in this category of needs.

Moreover, the psychological needs were most influenced by the level of anxiety (p < 0.01), fatigue (p < 0.01), and physical function (p < 0.05), and the three factors were responsible for 30.2% of the variance in this category of needs. The predictors of physical and daily living needs were anxiety (p < 0.01), fatigue (p < 0.01), social functioning (p < 0.01), role functioning (p < 0.05), and diarrhoea symptoms (p < 0.05). These five factors accounted for 34.4% of the physical and daily living needs variance. Thus, the regression analysis indicates that psychological aspects such as anxiety, cancer-related symptoms such as fatigue and diarrhoea, and emotional, physical, social and role functioning are critical elements that influence unmet needs of patients with cancer. Interventions targeting these aspects have a good chance of moderating the perceived unmet needs of patients with cancer in Uganda.

## Discussion

The study explored the unmet needs and outcomes of psychological well-being and QoL of adult cancer patients at the Uganda Cancer Institute. Findings show that many patients experience anxiety and depression symptoms, with the highest unmet needs in physical/daily living, psychological support, and health systems/information. These unmet needs strongly correlate with symptoms like fatigue and anxiety as well as diminished emotional, physical, social and role functioning. By assessing unmet needs among Ugandan cancer patients, this research addresses a gap in cancer care services. Moreover, analysing the link between the needs and health outcomes provides data that is useful when developing targeted interventions in resource-limited settings.

This study found that Ugandan cancer patients have a high prevalence of unmet needs, especially in psychological support, health systems/information, and physical/daily functioning. In contrast to high-income countries, the rates of clinically significant anxiety and depression symptoms among cancer patients typically range from 20% to 30% [1]. Our sample’s abnormal anxiety (45.9%) and depression (35.9%) symptom rates are significantly higher**.** Contextual factors such as a lack of psychological support resources [14], cancer stigma [13,21,31], and the high symptom burden [32], could be responsible for this disparity. While the HADS is widely used, the relatively low Cronbach’s alpha for the depression subscale in this study (0.56) suggests that cultural or linguistic adaptation may be needed, and this challenge has also been reported in other African settings [33]. This limitation underscores the importance of contextually validating psychological measures to ensure accurate assessment of symptoms and the effects of interventions, especially in resource-limited settings such as Uganda.

Our finding that physical and daily living needs were most prominent is consistent with studies from other sub-Saharan African countries, which also report high levels of pain, fatigue, and functional impairment among cancer patients [15,34]. In contrast, studies from high-income settings often emphasize and found unmet needs in the areas of sexuality and patient care/support as more significant [35]. This indicates that prioritising basic symptom management and daily functioning is more essential in Uganda. Such a distinction may be influenced by cultural aspects, like a hesitance to talk about sexuality, alongside the significant effects of fundamental physical and informational needs in settings with limited resources.

Another significant finding is the high level of unmet needs related to health systems and information. Many patients reported difficulties in understanding their illness, treatment options, and prognosis, which can increase anxiety and decrease adherence to recommended therapies. This aligns with previous Ugandan and other African studies highlighting gaps in patient education and communication [13,36–38]. In contrast, robust patient navigation and education programs in high-income countries have been shown to reduce unmet informational needs and improve outcomes by reduce these unmet informational needs by coordinating care, facilitating communication between patients and providers, providing psychosocial support, and helping patients navigate complex health systems [39]. The results highlight the need for culturally and linguistically appropriate educational interventions within Uganda’s oncology care system.

Findings from both high-income and LMIC nations support the strong correlation between unmet demands and psychological discomfort (such as anxiety and depression) as well as a lower QoL [35]. However, the strength of these correlations in our research indicates that the effects of unmet needs would be much more pronounced in environments with underfunded healthcare systems and patients dealing with extra obstacles, including delayed diagnosis [40,41], financial difficulties [13,42], and restricted access to services such as palliative care [43]. For instance, the substantial correlation between unmet requirements and fatigue is consistent with research from Uganda and other African nations, where inadequate provider training and prescription shortages frequently result in inefficient symptom management.

The results demonstrate that unmet needs are significantly associated with impaired emotional, physical, social, and role functioning. This multifaceted impact is well-documented in the literature: unmet needs are strong predictors of poor quality of life and increased healthcare utilization in cancer populations globally [35]. In the Ugandan context, where social support systems may be strained and formal supportive care is limited, the consequences of unmet needs can be even more profound. The association between unmet needs and symptoms such as diarrhoea and fatigue highlights the interconnectedness of physical and psychosocial domains, reinforcing the call for a holistic approach to cancer care.

Our study employed standardized instruments (HADS, EORTC QLQ-C30, and the SCNS-SF-34), facilitating direct comparisons with global literature. However, the HADS-D subscale’s low Cronbach’s alpha (0.56) raises concerns regarding the reliability of depression measurement in this context. This limitation (also reported by studies in other settings in Africa) may indicate cultural differences in expressing or reporting depressive symptoms, potential translation issues, and the need for contextual adaptation of the tool.

Conclusion: The current study presents strong evidence that unmet needs exist every day among cancer patients in Uganda, and these seem to significantly affect their psychological well-being and quality of life. These findings emphasise the critical need for integrated, nurse-led supportive care programs focusing on symptom management, psychosocial support, and patient education.

Limitations: In addition to the cross-sectional design and reliance on self-reported data, this study’s single-centre, inpatient convenience sample from one site limits the generalizability of our findings. As a national referral and teaching hospital, UCI serves a specific patient population with access to certain cancer treatments and resources that may not be widely available in rural or other settings across Uganda or similar low-resource contexts. Consequently, the unmet needs and health outcomes observed may not fully represent the broader cancer patient population. This limitation affects the applicability of our results to other care settings. We recommend that future research include multiple and diverse clinical sites, including rural and outpatient populations, to validate and extend these findings.

Our analytic approach included the selection of predictors based on significant bivariate correlations to maintain model simplicity and avoid overfitting. However, this method may lead to residual confounding, as it was not possible to adjust for all potential confounders due to sample size and data constraints. We acknowledge this limitation and recommend that future studies with larger samples to explore with more comprehensive adjustments to better delineate independent predictors of unmet needs.

Clinical and Policy Implications: These findings highlight the critical importance of implementing integrated, nurse-led supportive care interventions for adult in patients with cancer at the Uganda Cancer Institute and comparable environments. To adequately address the unmet needs, the interventions must focus on thorough symptom management, psychosocial assistance, and patient education. It is vital to integrate routine screening for unmet needs and psychological distress into standard oncology care and establish referral pathways for individuals requiring specialized support. Additionally, enhancing health system capabilities through provider training, resource allocation, and creating culturally relevant educational materials is essential.

Future research should explore the unmet needs of cancer patients in outpatient and rural settings, where barriers may be even greater. Longitudinal studies are needed to assess the impact of targeted interventions on unmet needs, psychological symptoms, and quality of life. Validation of locally adapted assessment tools for psychological distress will also be critical to identify and address these issues accurately among cancer patients and their family caregivers.

## Supporting information

S1 Checklist Strobe Checklist. (PDF)

## References

[1] Sung H, Ferlay J, Siegel RL, Laversanne M, Soerjomataram I, Jemal A, et al. Global Cancer Statistics 2020: GLOBOCAN Estimates of Incidence and Mortality Worldwide for 36 Cancers in 185 Countries. CA Cancer J Clin. 2021;71(3):209–49. doi: 10.3322/caac.21660 33538338

[2] Lin L, Li Z, Yan L, Liu Y, Yang H, Li H. Global, regional, and national cancer incidence and death for 29 cancer groups in 2019 and trends analysis of the global cancer burden, 1990–2019. J Hematol Oncol. 2021;14:1–24. 34809683 10.1186/s13045-021-01213-z PMC8607714

[3] Ferlay J, Ervik M, Lam F, Colombet M, Mery L, Piñeros M, et al. Global cancer observatory: cancer today. Lyon, France: International Agency for Research on Cancer; 2018.

[4] Bukirwa P, Wabinga H, Nambooze S, Amulen PM, Joko WY, Liu B, et al. Trends in the incidence of cancer in Kampala, Uganda, 1991 to 2015. Int J Cancer. 2021;148(9):2129–38. doi: 10.1002/ijc.33373 33129228

[5] Jatho A, Mugisha NM, Kafeero J, Holoya G, Okuku F, Niyonzima N, et al. Capacity building for cancer prevention and early detection in the Ugandan primary healthcare facilities: Working toward reducing the unmet needs of cancer control services. Cancer Med. 2021;10(2):745–56. doi: 10.1002/cam4.3659 33319508 PMC7877353

[6] O’Brien M, Mwangi-Powell F, Adewole IF, Soyannwo O, Amandua J, Ogaja E, et al. Improving access to analgesic drugs for patients with cancer in sub-Saharan Africa. Lancet Oncol. 2013;14(4):e176–82. doi: 10.1016/S1470-2045(12)70343-1 23561749

[7] Ddungu H, Kumaketch E, Namisango E. Assessment of clinical and psychological needs of patients with metastatic breast cancer: challenges and gaps in meeting their needs in Uganda. American Society of Clinical Oncology; 2018.

[8] Bender Ignacio R, Ghadrshenas M, Low D, Orem J, Casper C, Phipps W. HIV Status and Associated Clinical Characteristics Among Adult Patients With Cancer at the Uganda Cancer Institute. J Glob Oncol. 2018;4:1–10. doi: 10.1200/JGO.17.00112 30241139 PMC6181185

[9] Low DH, Phipps W, Orem J, Casper C, Bender Ignacio RA. Engagement in HIV care and access to cancer treatment among patients with HIV-associated malignancies in Uganda. J Glob Oncol. 2019;5:1–8. 10.1200/JGO.18.00187 PMC6426497 30763144

[10] Uwayezu MG, Nikuze B, Maree JE, Buswell L, Fitch MI. Competencies for Nurses Regarding Psychosocial Care of Patients With Cancer in Africa: An Imperative for Action. JCO Glob Oncol. 2022;8:e2100240. doi: 10.1200/GO.21.00240 35044834 PMC8789211

[11] Uwayezu MG, Sego R, Nikuze B, Fitch M. Oncology nursing education and practice: looking back, looking forward and Rwanda’s perspective. Ecancermedicalscience. 2020;14:1079. doi: 10.3332/ecancer.2020.1079 32863873 PMC7434500

[12] Makumi D. Cancer Nurses in Africa Finding Their Footing. Asia Pac J Oncol Nurs. 2017;4(1):4–5. doi: 10.4103/2347-5625.199082 28217723 PMC5297230

[13] Nakaganda A, Solt K, Kwagonza L, Driscoll D, Kampi R, Orem J. Challenges faced by cancer patients in Uganda: Implications for health systems strengthening in resource limited settings. J Cancer Policy. 2021;27:100263. doi: 10.1016/j.jcpo.2020.100263 35559936

[14] Katende G, Nakimera L. Prevalence and correlates of anxiety and depression among family carers of cancer patients in a cancer care and treatment facility in Uganda: a cross-sectional study. Afr Health Sci. 2017;17(3):868–76. doi: 10.4314/ahs.v17i3.30 29085415 PMC5656193

[15] Muliira JK, Kizza IB. The other untold burden of cancer in sub-Saharan Africa: Anxiety and depressive symptoms among family caregivers of adult cancer patients. Int J Africa Nurs Sci. 2019;11:100166. doi: 10.1016/j.ijans.2019.100166

[16] Bray F, Parkin DM, African Cancer Registry Network. Cancer in sub-Saharan Africa in 2020: a review of current estimates of the national burden, data gaps, and future needs. Lancet Oncol. 2022;23(6):719–28. doi: 10.1016/S1470-2045(22)00270-4 35550275

[17] Kiwanuka F. Complementary and Alternative Medicine use: Influence of Patients’ Satisfaction with Medical Treatment among Breast Cancer Patients at Uganda Cancer Institute. Adv Biosci Clin Med. 2018;6(1):24. doi: 10.7575/aiac.abcmed.v.6n.1p.24

[18] Jermini M, Dubois J, Rodondi P-Y, Zaman K, Buclin T, Csajka C, et al. Complementary medicine use during cancer treatment and potential herb-drug interactions from a cross-sectional study in an academic centre. Sci Rep. 2019;9(1):5078. doi: 10.1038/s41598-019-41532-3 30911084 PMC6434040

[19] Low D, Merkel EC, Menon M, Lyman GH, Ddungu H, Namukwaya E, et al. Chemotherapy Use at the End of Life in Uganda. J Glob Oncol. 2017;3(6):711–9. doi: 10.1200/JGO.2016.007385 29244988 PMC5735970

[20] Harding R, Selman L, Agupio G, Dinat N, Downing J, Gwyther L, et al. The prevalence and burden of symptoms amongst cancer patients attending palliative care in two African countries. Eur J Cancer. 2011;47(1):51–6. doi: 10.1016/j.ejca.2010.08.003 20822896

[21] Meacham E, Orem J, Nakigudde G, Zujewski JA, Rao D. Exploring stigma as a barrier to cancer service engagement with breast cancer survivors in Kampala, Uganda. Psychooncology. 2016;25(10):1206–11. doi: 10.1002/pon.4215 27421234

[22] Fitch MI. Supportive care framework. Can Oncol Nurs J. 2008;18(1):6–24. doi: 10.5737/1181912x181614 18512565

[23] Faul F, Erdfelder E, Lang A-G, Buchner A. G*Power 3: a flexible statistical power analysis program for the social, behavioral, and biomedical sciences. Behav Res Methods. 2007;39(2):175–91. doi: 10.3758/bf03193146 17695343

[24] Annunziata MA, Muzzatti B, Bidoli E, Flaiban C, Bomben F, Piccinin M, et al. Hospital Anxiety and Depression Scale (HADS) accuracy in cancer patients. Support Care Cancer. 2020;28(8):3921–6. doi: 10.1007/s00520-019-05244-8 31858249

[25] Djukanovic I, Carlsson J, Årestedt K. Is the Hospital Anxiety and Depression Scale (HADS) a valid measure in a general population 65–80 years old? A psychometric evaluation study. Health Qual Life Outcomes. 2017;15:1–10. 28978356 10.1186/s12955-017-0759-9 PMC5628437

[26] Aaronson NK, Ahmedzai S, Bergman B, Bullinger M, Cull A, Duez NJ, et al. The European Organization for Research and Treatment of Cancer QLQ-C30: a quality-of-life instrument for use in international clinical trials in oncology. J Natl Cancer Inst. 1993;85(5):365–76. doi: 10.1093/jnci/85.5.365 8433390

[27] Naamala A, Eriksson LE, Orem J, Nalwadda GK, Kabir ZN, Wettergren L. Health-related quality of life among adult patients with cancer in Uganda. 2022. 10.1080/16549716.2024.2325728 PMC11008308 38596846

[28] Boyes A, Girgis A, Lecathelinais C. Brief assessment of adult cancer patients’ perceived needs: development and validation of the 34-item Supportive Care Needs Survey (SCNS-SF34). J Eval Clin Pract. 2009;15(4):602–6. doi: 10.1111/j.1365-2753.2008.01057.x 19522727

[29] Lee H, Jang Y, Jeong Y. Supportive Care Needs Survey: A reliability generalization meta-analysis. Palliat Support Care. 2023;21(4):714–26. doi: 10.1017/S1478951522001791 36779271

[30] Han Y, Zhou Y, Wang J, Zhao Q, Qin H, Fan Y, et al. Psychometric testing of the Mandarin version of the 34-item Short-Form Supportive Care Needs Survey in patients with cancer in mainland China. Support Care Cancer. 2017;25(11):3329–38. doi: 10.1007/s00520-017-3750-4 28551842

[31] Nabisubi P, Nanyingi M, Okeny PK. Lived experiences of prostate cancer patients below 55 years of age: A phenomenological study of outpatients receiving treatment at the Uganda cancer Institute. 2020.

[32] Zirimenya L, Musoke C, Hutt E. Symptom Prevalence and Burden in Cancer Patients with and without HIV/AIDS Reffered for Palliative Care. JHS. 2015;3(6). doi: 10.17265/2328-7136/2015.06.006

[33] Abiodun OA. A validity study of the Hospital Anxiety and Depression Scale in general hospital units and a community sample in Nigeria. Br J Psychiatry. 1994;165(5):669–72. doi: 10.1192/bjp.165.5.669 7866683

[34] Bray F, Parkin DM, African Cancer Registry Network. Cancer in sub-Saharan Africa in 2020: a review of current estimates of the national burden, data gaps, and future needs. Lancet Oncol. 2022;23(6):719–28. doi: 10.1016/S1470-2045(22)00270-4 35550275

[35] Bergerot C, Jacobsen PB, Rosa WE, Lam WWT, Dunn J, Fernández-González L, et al. Global unmet psychosocial needs in cancer care: health policy. eClinicalMedicine. 2024;78:102942. doi: 10.1016/j.eclinm.2024.102942 39634034 PMC11615525

[36] Mwaka AD, Okello ES, Wabinga H, Walter FM. Symptomatic presentation with cervical cancer in Uganda: a qualitative study assessing the pathways to diagnosis in a low-income country. BMC Womens Health. 2015;15:15. doi: 10.1186/s12905-015-0167-4 25783641 PMC4337099

[37] Esther N, Julius S, Deogratius MA. Understanding health-seeking and adherence to treatment by patients with esophageal cancer at the Uganda cancer Institute: a qualitative study. BMC Health Serv Res. 2021;21(1):159. doi: 10.1186/s12913-021-06163-3 33602201 PMC7890846

[38] Kagimu R. Information needs, access and coping strategies for cervical cancer clients at Mulago Uganda Cancer Institute. J Health Informat Africa. 2019;6(2):45–50.

[39] Chen M, Wu VS, Falk D, Cheatham C, Cullen J, Hoehn R. Patient Navigation in Cancer Treatment: A Systematic Review. Curr Oncol Rep. 2024;26(5):504–37. doi: 10.1007/s11912-024-01514-9 38581470 PMC11063100

[40] Farmer P, Frenk J, Knaul FM, Shulman LN, Alleyne G, Armstrong L, et al. Expansion of cancer care and control in countries of low and middle income: a call to action. Lancet. 2010;376(9747):1186–93. doi: 10.1016/S0140-6736(10)61152-X 20709386

[41] James ND, Tannock I, N’Dow J, Feng F, Gillessen S, Ali SA, et al. The Lancet Commission on prostate cancer: planning for the surge in cases. Lancet. 2024;403(10437):1683–722. doi: 10.1016/S0140-6736(24)00651-2 38583453 PMC7617369

[42] Gordon LG, Merollini KMD, Lowe A, Chan RJ. A Systematic Review of Financial Toxicity Among Cancer Survivors: We Can’t Pay the Co-Pay. Patient. 2017;10(3):295–309. doi: 10.1007/s40271-016-0204-x 27798816

[43] Reid E, Lukoma M, Ho D, Bagasha P, Leng M, Namukwaya L. Palliative care needs and barriers in an urban Ugandan Emergency Department: A mixed-methods survey of emergency healthcare workers and patients. Afr J Emerg Med. 2023;13(4):339–44. doi: 10.1016/j.afjem.2023.11.005 38162896 PMC10757186

