# Supplementary material for: Unmet needs and quality of life of adult patients while receiving cancer treatment in Uganda: A cross-sectional study
Source: PLoS One. 2025 Dec 31;20(12):e0339827. doi: 10.1371/journal.pone.0339827 (PMC12755768; doi:10.1371/journal.pone.0339827)
Supplement: S1 Checklist — (PDF) [file pone.0339827.s001.pdf]

Item No.	STROBE Item Description	Manuscript Location (Page, Line)
Title and Abstract		
1a	Indicate the study design with a commonly used term in the title or abstract	Page 1 Line 2
1b	Provide in the abstract an informative and balanced summary of what was done and what was found	Page 2, lines 26-44
Introduction		
2	Background/rationale: Explain the scientific background and rationale for the investigation being reported	Page 3 Lines 49-103
3	Objectives: State-specific objectives, including any prespecified hypotheses	Page 5, lines 101-102
Methods		
4	Study design: Present key elements of the study design early in the paper	Page 5, line 107
5	Setting: Describe the setting, locations, and relevant dates, including periods of recruitment, exposure, follow-up, and data collection	Page 5 line 112-114
6a	Participants: Give the eligibility criteria, and the sources and methods of selection of participants	Page 5 Lines 112-114
6b	For matched studies, give the matching criteria and the number of exposed and unexposed.	Not applicable (cross-sectional, not matched)
7	Variables: Clearly define all outcomes, exposures, predictors, potential confounders, and effect modifiers. Give diagnostic criteria, if applicable	Page 7 lines 141-142
8	Data sources/measurement: For each variable of interest, give sources of data and details of methods of assessment (measurement). Describe comparability of assessment methods if there is more than one group	Page 7 Lines 141-195
9	Bias: Describe any efforts to address potential sources of bias	Page 6 Lines 121-139
10	Study size: Explain how the study size was arrived at	Page 6 Lines 121-128

11	Quantitative variables: Explain how quantitative variables were handled in the analyses. If applicable, describe which groupings were chosen and why	Page 7-8 Lines 152-196
12a	Statistical methods: Describe all statistical methods, including those used to control for confounding	Page 7-8 Lines 152-196
12b	Describe any methods used to examine subgroups and interactions	Page 7-8 Lines 152-196
12c	Explain how missing data were addressed	N/A
12d	If applicable, describe analytical methods taking account of sampling strategy	Not applicable (consecutive sampling, no weighting)
12e	Describe any sensitivity analyses	Not reported
Results		
13a	Participants: Report numbers of individuals at each stage of study e.g., numbers potentially eligible, examined for eligibility, confirmed eligible, included in the study, completing follow-up, and analysed	Page 6 lines 126-128
13b	Give reasons for non-participation at each stage	Page 6 Lines 137-139 Exclusion criteria given
13c	Consider use of a flow diagram	Not included
14a	Descriptive data: Give characteristics of study participants (e.g., demographic, clinical, social) and information on exposures and potential confounders	Page 12-13 Lines 277-279
14b	Indicate number of participants with missing data for each variable of interest	Not explicitly stated
15	Outcome data: Report numbers of outcome events or summary measures	Page 15 Line 288, Page 16 line 323
16a	Main results: Give unadjusted estimates and, if applicable, confounder-adjusted estimates and their precision (e.g., 95% confidence interval). Make clear which confounders were adjusted for and why they were included	Page 19-20 Lines 350-352
16b	Report category boundaries when continuous variables were categorized	Pages 13 and 14 in Tables 1 and 2 ,
16c	If relevant, consider translating estimates of relative risk into absolute risk for a meaningful time period	Not applicable (cross-sectional, not longitudinal)

17	Other analyses: Report other analyses done e.g., analyses of subgroups and interactions, and sensitivity analyses	no sensitivity analyses reported
Discussion		
18	Key results: Summarise key results with reference to study objectives	Page 21 lines 357-364
19	Limitations: Discuss limitations of the study, taking into account sources of potential bias or imprecision. Discuss both direction and magnitude of any potential bias	Page 23-24 lines 422-427
20	Interpretation: Give a cautious overall interpretation of results considering objectives, limitations, multiplicity of analyses, results from similar studies, and other relevant evidence	Page 21-23 lines 365-421
21	Generalisability: Discuss the generalisability (external validity) of the study results	Page 8, Lines 170-172<br>Page 21, Lines 365-377<br>Page 4, Lines 83-88<br>Page 12, Lines 256-264<br>Page 7, Lines 137-139<br>Page 24, Lines 422-427
Other information		
22	Funding: Give the source of funding and the role of the funders for the present study and, if applicable, for the original study on which the present article is based	Page 25, Lines 445–446
